# *Candida albicans* and non-*albicans* Isolates from Bloodstream Have Different Capacities to Induce Neutrophil Extracellular Traps

**DOI:** 10.3390/jof5020028

**Published:** 2019-04-01

**Authors:** Lizbeth Campos-Garcia, Rocio Jimena Jimenez-Valdes, Romel Hernandez-Bello, Jose Palma-Nicolas, Gloria Maria Gonzalez, Alejandro Sanchez-Gonzalez

**Affiliations:** 1 Departamento de Microbiología, Facultad de Medicina, Universidad Autónoma de Nuevo León, Monterrey 64460, Mexico; qcblizbeth@gmail.com (L.C.-G.); romelhdezb.d@gmail.com (R.H.-B.); palmanicolasjp@gmail.com (J.P.-N.); gloria62@hotmail.com (G.M.G.); 2 Unidad Monterrey, Centro de Investigación y de Estudios Avanzados del Instituto Politécnico Nacional, Vía del Conocimiento 201, Parque PIIT, Apodaca, Monterrey 66628, Mexico; rjjv17@gmail.com

**Keywords:** neutrophil extracellular traps, *Candida albicans*, *Candida* non-*albicans*, phospholipases

## Abstract

Neutrophils activated with pathogens or their products induce formation of extracellular traps (NETs), but if this constitutes a general response against all pathogenic species in a single genus or intrageneric differences exist remains unknown, yet this is of great importance for the establishment of effective treatments. To determine this, we analyzed neutrophil extracellular traps formation after the stimulation with bloodstream isolates from different *Candida* species (*Candida albicans*, *C. tropicalis*, *C. parapsilosis*, and *C. glabrata*), and found that each species has a different capacity to induce DNA extrusion, which is independent of their morphology (yeast or hyphae). We observed that phospholipase producer’s strains and their secretion products were able to induce NETs, a property not observed with phospholipase deficient strains, with exception of some *Candida glabrata* sensu stricto isolates, which showed no NETs induction although they did show phospholipase production. To further analyze this, we extended our study to include *Candida glabrata* cryptic species (*C. bracarensis* and *C. nivariensis*) and no extracellular traps formation was observed. Here, we contribute to the understanding of how neutrophils initiate NETs, and we found that certain strains may have a differential capacity to trigger these structures, which may explain the high mortality of some isolates.

## 1. Introduction

Neutrophils are the first immune cells recruited to an infection site where they recognize and kill the infectious agents by phagocytosis, degranulation, and extracellular traps (NETs) [1,2,3,4,5]. NETs are composed of modified chromatin decorated with specific cytoplasmic and granular proteins which entrap and eliminate bacteria, fungi, parasites, and virus. The formation of NETs is characterized by the activation of the NADPH oxidase that leads to ROS formation which in turn activate the neutrophil granules myeloperoxidase (MPO) and neutrophil elastase (NE) promoting the translocation of NE to the nucleus which cleaves histones leading first to the fusion of the nuclear lobules, and later to the decondensation of nuclear material. In this stage, the chromatin is modified and mixed with granule and cytoplasmic proteins and eventually extruded from neutrophils [6]. The importance of NETs in host defense against fungi is underscored by observations made in chronic granulomatous disease patients. These patients present recurrent infections because they carry mutations in the NADPH oxidase showing a reduced antimicrobial activity due to its inability to produce reactive oxygen species (ROS) and diminished NETs formation [7]. Moreover, gene therapy for NETs formation restoring, have showed impressive effects on patients infected with *Aspergillus nidulans*, a microbe not susceptible to phagocytosis [8]. 

Fungal diseases and in particular those associated with the *Candida* complex (*C. albicans*, and non- *albicans* such as *C. glabrata*, *C. parapsilosis*, and *C. tropicalis*) are now considered a global threat as they constitute an important cause of nosocomial infections of the blood stream with high mortality rates [9]. Therefore, studies focused on the understanding of the immune response against the *Candida* complex are relevant. Among the immune responses elicited by *Candida albicans*, the formation of neutrophil extracellular traps by the host has been documented; it has been demonstrated that calprotectin-rich NETs can restrict *Candida albicans* infections by preventing their spread and promoting their death [10]. However, there are no studies focused on determining if the formation of NETs constitutes a general response against all species of the *Candida* complex.

Efforts have been made to determine the fungal molecules and the receptors involved in the formation of the NETs. Some groups observed the formation of NETs in the presence of hyphae of *C. albicans*, but not by yeast; these descriptions led them to propose a model in which neutrophils are able to ‘detect’ the size of the fungi through the Dectin-1 receptor and, therefore, if the pathogen is large and cannot be eliminated by phagocytosis then the NETs are induced [11]. The importance of Dectin-1 receptor for fungi recognition by neutrophils has already been partially characterized, as it was demonstrated that NETs are induced to solve the infection by *Paracoccidioides brasiliensis* after the interaction with this receptor [12]. Studies aimed at discovering fungi molecules involved in NETs induction have described that the β-glucan and mannans can initiate rapid extrusion of these structures, as well as secreted aspartic proteases (4 and 6) after interaction with CD16, CD18 and Dectin-1 in the neutrophil membrane [13,14,15]; however, the participation of additional factors involved in fungi virulence that can be recognized by neutrophils to promote NETs cannot be excluded due to the active secretion and expression of these factors during the infection and the establishment of these pathogens.

Among the molecules that are known to actively participate in various aspects of virulence by pathogenic fungi are the phospholipases. Increased expression of phospholipases A, B, and C in yeast and hyphae and active secretion in the extracellular environment during tissue invasion have been associated with increased virulence of *Candida albicans* [16,17,18,19,20,21]. Although the participation of phospholipases has not been related to the formation of NETs, some evidence could suggest their involvement; as it is known that fungal phospholipases can degrade glycerophospholipids in cell membranes, promoting the activation of the arachidonic acid pathway, which, in turn, activates protein kinase C (PKC) to promote inflammation [22,23]. This is interesting, since it has been reported that PKC activation is a primordial step that leads to NETs formation [24,25,26]. From these observations, it is possible that fungal phospholipases may be a stimulus recognized by neutrophils for NETs induction. Therefore, studies aimed at characterizing their kinetics of production in clinical isolates and their role in NETs formation, and if differences exist among species of the *Candida* complex could contribute to the understanding of the response of the immune system against the species that belong to these pathogens.

Here, we analyzed the capacity of clinical isolates of *Candida albicans* and non-*albicans* to induce NETs formation, and we observed that each species has a different capacity to induce these structures. Additionally, we studied the phospholipase production among these isolates and found a strong correlation in their production with the NETs induction.

## 2. Materials and Methods

### 2.1. Ethics Statement

This protocol followed the guidelines for ethical practices with human subjects and was approved (Protocol No. MB15-003) by the Research Ethics Committee of the Medicine Faculty and University Hospital of the Autonomous University of Nuevo León, Mexico. The volunteer donors (20 different donors), signed an informed consent for blood donation and participation in this research. 

### 2.2. Candida spp. Strains

All used strains of *Candida* spp. were obtained from bloodstream and isolated in blood cultures at the Regional Center for the Control of Infectious Diseases (CERCEI) of the Faculty of Medicine of the Autonomous University of Nuevo Leon. 30 isolates of *C. albicans*, 30 isolates of *C. parapsilosis*, 30 isolates of *C. tropicalis*, 30 isolates of *C. grabrata* sensu stricto, and one isolate each of *C. bracarensis* and *C. nivariensis* were grown on Sabouraud dextrose agar (BD, Sparks, MD) for 24 h–48 h at 30 °C. To differentiate *C. albicans* from other *Candida* species (*C. parapsilosis*, *C. tropicalis*, *C. glabrata* sensu stricto, *C. bracarensis*, and *C. nivariensis*), chlamydospore formation and filamentation assay primary tests were carried out [27]. To fully identify non-*Candida* strains and to confirm those of *C. albicans*, the API 20C AUX system (bio Mérieux, France) was used, following manufacturer’s protocols. 

### 2.3. Candida spp. Growth Determination

To determine the growth on liquid, *Candida* spp. strains were inoculated in Sabouraud dextrose broth for 24 h, 72 h, or 120 h at 37 °C, and then cultivated in Sabouraud dextrose agar plates. Samples were collected every 24 hours for 96 h and centrifuged at 3000× *g* for 5 min. Supernatants were collected, filtered using 0.20 μm syringe filters (Corning, NY, USA), quantified and stored at −20 °C for future use. On the other hand, the pellet was suspended in sterile water; optical density was measured (600 nm) and adjusted to 1 OD (±3 × 10^7^ fungi per mL). To determine growth on agar plates, fungi were inoculated on Sabouraud dextrose agar, and then colonies were collected at indicated time points and suspended in sterile water, measured at 600 nm, and adjusted to 1 OD by serial dilutions. Additionally, to confirm yeast count and to minimize the effect that could arise in the optical density’s measurements due to yeast size among the isolates, some of the adjusted samples were taken, labeled with a solution of propidium iodide (5 mM), and counted on a Neubauer chamber (Propper, NY, 11101) using a fluorescence microscope (Zeiss Axioplan, West Germany).

### 2.4. DNAse and Hemolysin Activity in Candida spp. Isolates

To determine DNAse activity, strains were inoculated directly on DNAse test agar with methyl green medium (Becton Dickinson, France), and incubated for 7 days at 37 °C. For hemolysin determination, strains were inoculated in Sabouraud blood medium (Sabouraud agar 65g/L, 3% w/v glucose and 7% v/v blood). *Staphylococcus aureus* ATCC 25923 was used as a positive control. The activity was determined by measuring the halo of decoloration in the medium (DNAse) or the hemolysis halo (hemolysins) around the fungal colony, a quotient < 0.69 corresponds to a high activity (represented as ++++), 0.70 to 0.79 moderate activity (represented as +++), 0.80 to 0.89 poor activity (represented as ++), 0.90 to 0.99 weak activity (represented as +), and 1 refers to negative activity (represented as −).

### 2.5. Production Kinetics and Determination of Activity of Fungal Phospholipases

To determine phospholipase production and activity of fungal phospholipases, *Candida* spp. were grown for 24 h, 72 h, or 120 h on Sabouraud dextrose agar or broth, diluted and adjusted to 1 × 10^7^ CFU/mL, followed by seeding on Sabouraud dextrose agar with egg yolk (BD Difco, Franklin Lakes, NJ, USA), and incubated at 37 °C. Phospholipase activity (Pz index) was determined by measuring the precipitation halo around the growing colony indicative of enzyme activity over the phosphatidylcholine contained in the medium (a quotient < 0.69 corresponds to a very strong activity, 0.70 to 0.79 strong activity, 0.80 to 0.89 mild activity, 0.90 to 0.99 weak activity, and 1 refers to negative activity), as has been previously described [28,29]. As negative control and for comparative purposes, we used a phospholipase deficient strain of *Trichosporum asahii* (ATCC 90039) [30]. Readings of the precipitation halos were taken every 24 h for 96 h.

### 2.6. Phospholipase Identification

Phospholipase species in yeast and in supernatant were identified by dot blot and western blot. For dot blot, total proteins from *Candida* spp. growth by 96 h on Sabouraud dextrose broth supplemented with egg yolk were obtained by sonication and detergent extraction and quantified using the Lowry method. From each sample, 200 μg of protein was probed on spots over PVDF membranes and allowed to dry at 37 °C for 1 h. After, tris buffered saline with Tween 20 + 5% non-fat milk (Sigma-Aldrich) was added for 1 h at room temperature followed by overnight incubation with anti-PLA2 (EPR8650) (1:1000), anti-PLB1 (ab92915) (1:4000), anti-PLCG2 (EPR1403) (1:10,000), or anti-PLD4 (ab137955) (1:500) (all from Abcam). After three washes, horseradish peroxidase conjugated secondary antibodies (1: 20,000; Jackson Immuno Research Laboratories, Inc.) were added and incubated for 1 h at room temperature. Blots were revealed with Pierce ECL western blotting Substrate (Rockford, IL); spot intensity was normalized against negative control and quantified by densitometry using ImageJ software. To identify phospholipases in supernatant, *Candida* spp. was grown at 37 °C for 48 h on Saboraud dextrose broth supplemented with 8% egg yolk, 4% glucose and 2% human serum. Yeast were pelleted by centrifugation and supernatant was taken and quantified by Lowry, samples were mixed with SDS loading buffer and boiled for 5 minutes, after 30 μg protein was resolved in 10% SDS-PAGE and transferred to PVDF membranes. Membranes were blocked for 1 h at room temperature with 5% non-fat milk + TBST (0.1% Tween 20), followed by overnight incubation with the phospholipase’s antibodies mentioned above. After three washes, horseradish peroxidase conjugated secondary antibodies were added (same as above, 1:20,000) and incubated for 1 h at room temperature. Finally, blots were developed by chemiluminescence with Pierce ECL Western Blotting Substrate.

### 2.7. Neutrophil Isolation

Blood was draw from healthy volunteers (20 different donors) in EDTA tubes (BD vacutainer, Franklin Lakes, NJ, USA), and neutrophils were isolated over Histopaque 1119 (Sigma-Aldrich, St. Louis, MO), followed by centrifugation on a discontinuous Percoll gradient (Sigma-Aldrich, St. Louis, MO, USA), as previously described [31]. Neutrophils were maintained on RPMI 1640 (phenol red-free) supplemented with 10 mM Hepes and 0.5% human serum albumin and quantified in Neubauer chamber. Cells displayed at least 95% viability (trypan blue exclusion test) before starting the experiment.

### 2.8. NETs Induction

The experiments were carried out with *Candida* spp. in the phase in which the maximum phospholipase activity was detected for each strain, which correspond to 96 h. To induce NETs with yeast-free supernatants, the fungi were grown for 96 h in Saboureau dextrose broth supplemented with egg yolk and on the day of the experiment, fungal media was collected, filtered (0.22 µM pore size) and then added to the cells at 4% final concentration. Neutrophils (2 × 10^5^ per well) were stained with 1 μg/mL of Hoechst and seeded on 24-well plates in RPMI 1640 supplemented with 10 mM HEPES and 0.5% human serum albumin and allowed to adhere for 30 min at 37 °C. *Candida* spp. (five representative strains of each species were selected and screened in their capacity to induce NETs; MOI 0.2:1), fungi culture medium filtered through 0.22 µM pore size (1 μg/mL), 40 nM PMA (Sigma Aldrich St. Louis, MO, USA), or sterile Trypticase soy broth (TSB) were added to the cells. Plates were centrifuged at 800× *g* for 5 min to increase the contact of fungi or proteins with the cells and incubated for 3 h at 37 °C. NETs formation was determined by stain the extracellular DNA with 5 μM Sytox Green (Invitrogen) for 10 min in the dark at room temperature. Images were taken with a fluorescence microscope (Zeiss Axioplan, West Germany) with 20× objectives. All experiments were performed in triplicate with neutrophils obtained from two different donors each time.

### 2.9. NETs Quantification

NETs were induced with PMA, *Candida* spp., or filtered medium, and extracellular DNA was stained Sytox Green. To determine the amount of NETs by nuclear area expansion, a protocol previously described was followed; this method is based on the changes in nuclear size and neutrophil shape during NETs formation. It has been observed that activated neutrophils undergoing NETs formation for 240 minutes possess a higher nuclear area, with a range between 120 and 350 µm^2^; in contrast, the normal average area of an untreated neutrophil is calculated to be ±80 µm^2^, assuming a circular shape, and using the formula π × r^2^ after Sytox green stain [6,31]. For this, micrographs were taken in a Zeiss Axiovert inverted epifluorescence microscope with 20× objective lenses. On these, a total of 600 cells per treatment were analyzed using ImageJ software, and the distribution of the number of cells across the range of nuclear area was obtained using the frequency function in Microsoft Excel. The Sytox-positive counts were divided by the total number of cells, determined from corresponding phase contrast images (Sytox/Total cells), and plotted as the percentage of Sytox-positive cells within each area range.

### 2.10. NETs Characterization

Untreated, PMA or *Candida* ssp. treated neutrophils (2 x 10^5^ per condition) were fixed with 4% paraformaldehyde, permeabilized (0.5% triton X-100) for 15 minutes and blocked with 3% BSA. After washing, the cells were stained for 1 h at 37 °C with anti-myeloperoxidase (1:400) (Dako, Agilent Technologies, Carpinteria, USA) and anti-calprotectin (clone MAC 387; 1:400) (abcam). Two more washes were made, followed by addition of secondary antibodies coupled to Alexa 568 and Alexa 488 (Molecular Probes) and incubated for 1 h at 37 °C. Next, cells were counterstained with ProLong Gold antifade reagent with DAPI (P36941; Molecular Probes). Epifluorescence images were captured using a Zeiss Axioplan upright epifluorescence microscope with 100× objectives and images were captured using a Media Cybernetics Evolution MP camera and Q Imaging software.

### 2.11. Statistical Analysis

Data were analyzed in Graph Pad Prism 6 software using ANOVA and Tukey´s variance analysis and two-way ANOVA with multiple comparison test of Sidak.

## 3. Results

### 3.1. NETs Induction by Clinical Isolates of *Candida*

**Figure 1:**
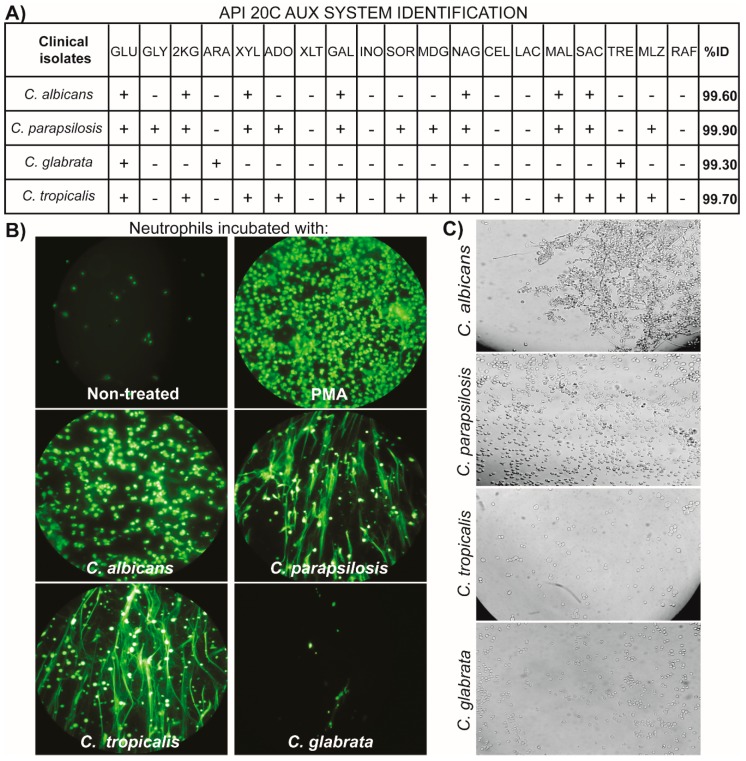
*Candida* isolates can promote NETs formation regardless its capacity to form filamentous structures. (**A**) Identification profiles of *Candida* isolates through their ability to use carbon substrates by API 20C AUX. (**B**) Neutrophils were incubated with *C. albicans*, *C. parapsilosis*, *C. tropicalis*, or *C. glabrata* isolates (MOI 0.2:1), PMA (40 nM) or non-treated (NT). Sytox Green was added after 180 min of incubation to detect extracellular DNA. A representative image of NETs induced by each strain is shown. (**C**) Filamentous structures are observed in *Candida albicans* isolates, but not in those of *C. parapsilosis*, *C. tropicalis*, or *C. glabrata*. A representative image of each species is shown.

Species identification using the API 20C AUX system confirmed the presence of *Candida albicans* and non - *albicans* strains (Figure 1A). Neutrophils incubation with *C. albicans*, *C. parapsilosis* and *C. tropicalis* induced NETs comparable to those induced by PMA. In contrast, cells incubated with *C. glabrata* did not induced any detectable levels of NETs (Figure 1B).

We also analyzed the morphology of each of our isolates. Only *C. albicans* was able to form filamentous structures; in contrast, neither *C. glabrata*, nor *C. parapsilosis*, nor *C. tropicalis* could form these structures (Figure 1C).

### 3.2. NETs Characterization

**Figure 2:**
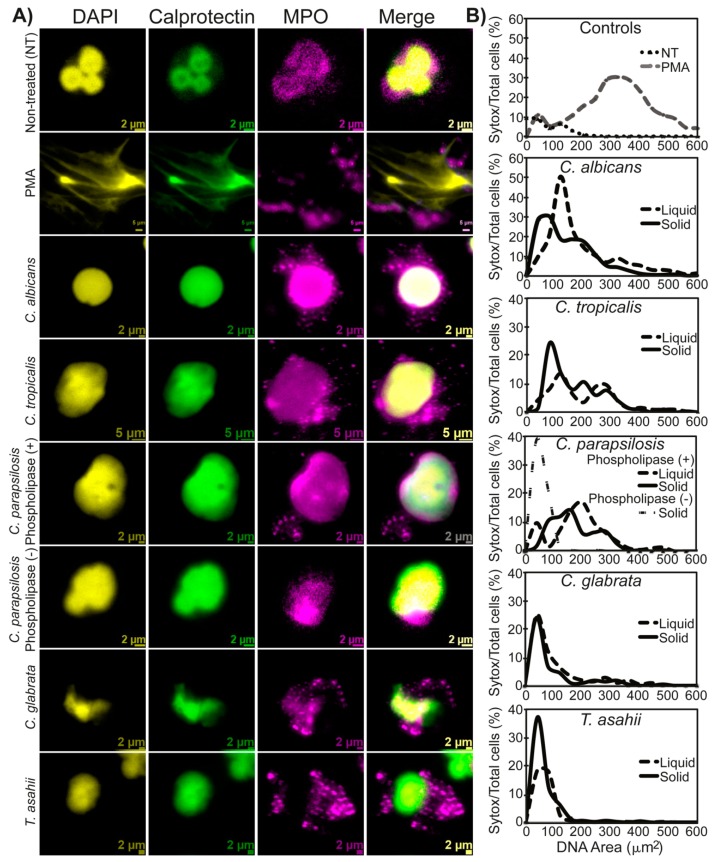
Nuclear changes and granule migration reminiscent of NETs occurred in neutrophils after treatment with *C. albicans*, *C. tropicalis*, and *C. parapsilosis* phospholipase positive, but not after incubation with *C. glabrata* or *C. parapsilosis* phospholipase negative strains. (**A**) Immunofluorescence microscopy images of neutrophils stained with myeloperoxidase (MPO) and calprotectin after treatment with PMA (positive control), non-treated (NT) or treated with *C. albicans*, *C. tropicalis*, *C. glabrata*, or with strains of *C. parapsilosis* producers of phospholipase or with phospholipase negative strains. In addition, for comparative purposes, neutrophils were incubated with a phospholipase negative strain of *Trichosporon asahii* for 180 min. To assess the nuclear morphology, the nucleus was stained with DAPI. From left to right, columns correspond to DAPI, calprotectin, MPO, and a merged image. (**B**) Nuclear area quantification of non-treated (NT) neutrophils or incubated with *C. albicans*, *C. parapsilosis*, *C. tropicalis*, *C. glabrata*, and *Trichosporon asahii* isolates grown on solid or liquid medium or PMA alone as a positive control. Additionally, a nuclear area quantification of neutrophils incubated with phospholipase-producing *C. parapsilosis* (+) or with a negative phospholipase strain (–) is shown. The nuclear area of neutrophils after stimulation was calculated as indicated in the Materials and Methods section, and the value corresponding to a given nuclear area range was plotted against the percentage of Sytox positive cells. Results are representative of three independent experiments and each condition was tested in triplicate.

We obtained different DNA extrusion morphologies (fibers or expanded nucleus), this may be due to the clinical isolates that we used. We next characterized NETs formation by calprotectin and myeloperoxidase (MPO) localization by immunofluorescence and nuclear area expansion quantification since NETs represent an ordered process in which the neutrophil granules promote nuclear delobulation followed by expansion. Our results show that neutrophils presented a delobulated nucleus and perinuclear granule localization, corresponding to the first stages of NETs formation after incubation with *C. albicans*, *C. tropicalis*, *C. parapsilosis*, or PMA. Conversely, after incubation with *C. glabrata*, calprotectin and MPO were not mobilized from its granule compartment and the nucleus did not increase its size (Figure 2A).

NETs were also quantified through determination of the nuclear area expansion. Cells incubated with *C. albicans*, *C. tropicalis*, and *C. parapsilosis* induced nuclear area expansion, although the nucleus expanded to a lesser extent than that observed after the stimulation with PMA. Moreover, and accordingly to our previous results, incubation with *C. glabrata* did not induce delobulation or an increase in the nuclear area, therefore remaining of equal size to that observed in untreated cells (Figure 2B). These results showed that either delobulated or expanded nuclear phenotypes are reminiscent of NETs.

### 3.3. Fungal Phospholipases Production and NETs Induction

**Figure 3:**
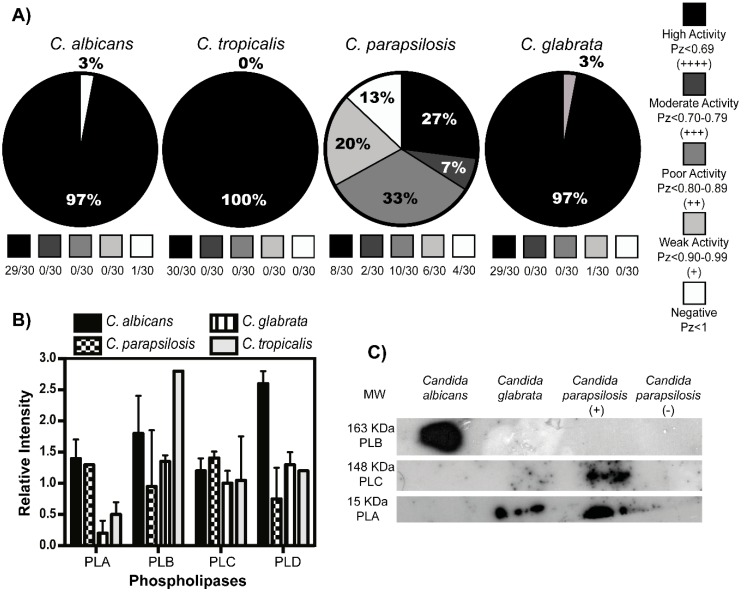
Phospholipase activity in the *Candida* isolates. (**A**) Isolates of each strain were grown on egg yolk medium. Phospholipase enzyme activity was determined by a Pz based index as described on Materials and Methods. Results are representative of three independent experiments in triplicate for each strain. (**B**) Clinical isolates of *Candida* ssp. produce phospholipases A, B, C, and D subtypes. The total protein from *Candida* ssp. strains on exponential growth phase was obtained and blotted in spots, followed by recognition of phospholipase subtypes with specific antibodies. The relative intensity of each spot was calculated by densitometry. Data is representative of three independent experiments in triplicate for each strain. (**C**) Clinical isolates of *Candida* secreted phospholipases. Yeast-free medium from strains on exponential phase growth was obtained, the proteins were quantified resolved by SDS-PAGE and blotted in nitrocellulose followed by the identification of the secreted phospholipases with monoclonal antibodies. The membranes were developed by chemiluminescence and the results are shown.

We sought to characterize the phospholipase activity in our *Candida* isolates. A very high enzymatic activity, ranging from 97 to 100% with marked, double, translucent, or small halo morphologies was observed for *C. albicans*, *C. tropicalis*, and *C. glabrata*. In contrast, isolates of *C. parapsilosis* presented a high variation in their enzymatic activities, ranging from high (27%), moderate (7%), weak (20%), poor (33%), or even negative (13%) and with large, small, or absent halo morphologies (Figure 3A and Supplementary Figure S1).

Phospholipases identification showed that the four subtypes (A, B, C, and D) are produced in the yeasts of all species (Figure 3B); however, the analysis of the secreted phospholipases demonstrated some differences. *C. albicans* secreted phospholipase B, whereas phospholipase-producing strains of *C. parapsilosis* showed secretion of mainly phospholipase A and C, in addition, *C. glabrata* species secreted low levels of phospholipase A. On the other hand, and as was expected, there was no secretion of phospholipases in phospholipase-negative strains of *C. parapsilosis* (Figure 3C).

**Figure 4:**
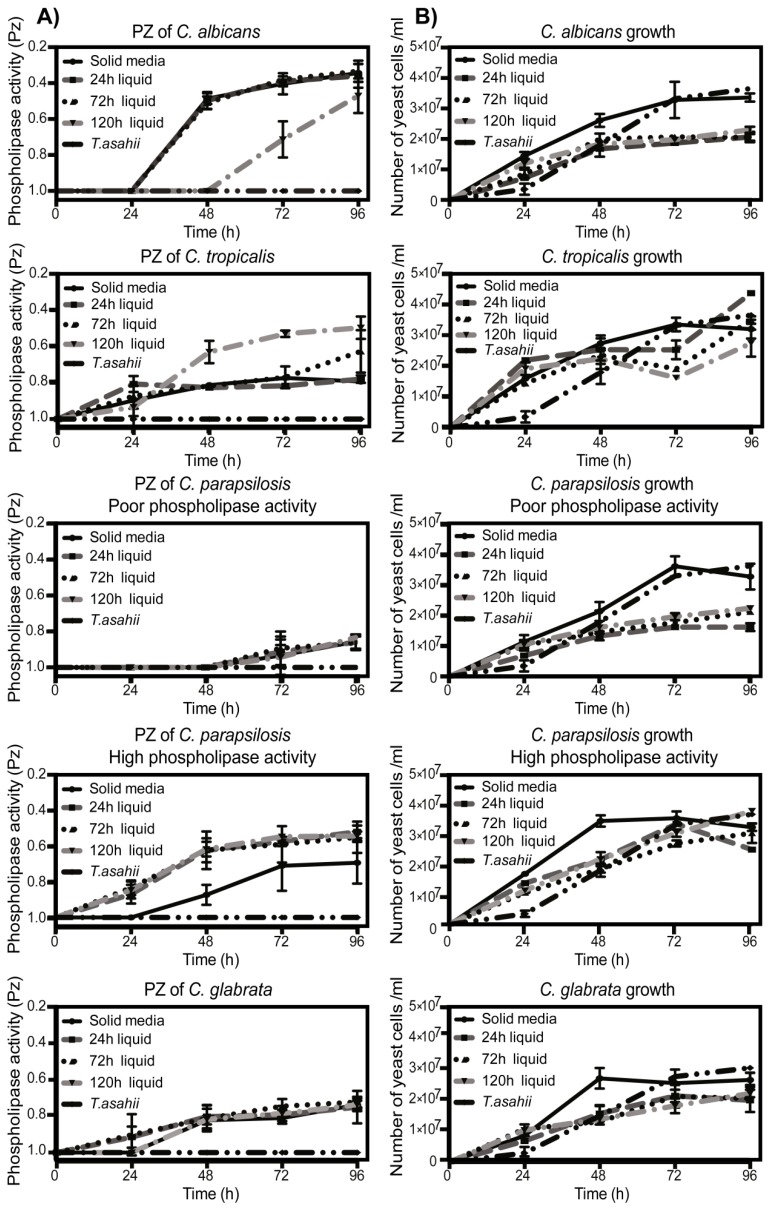
Growth conditions modify phospholipases production kinetics in *Candida* spp. *Candida* spp. were grown on solid medium without pre-culture, or were pre-cultured in liquid medium for 24, 72, or 96 h, after which growth was measured at 600 nm, and the amount of fungi was adjusted to 1 OD and then seeded on plates containing phospholipases substrate to determine their respective phospholipase activity (**A**, left column) by measuring the size of the precipitation halo of activity (Pz index), and growth rate (**B**, right column) by measuring the total diameter of the colony. Precipitation halos measurements were taken every 24 h for 4 days. Results are representative of three independent.

Of importance for our study is that all the strains used are blood isolates from systemic infections, because of this we were interested in studying if the environment in which the fungus is grown could influence the expression of these enzymes. To analyze this, we carried out experiments in which each strain was grown in liquid or solid medium for 24, 72, or 96 h. We observed that phospholipase activity of *C. albicans* was mostly not affected, and some differences could only be observed in older cultures in which after an initial delay in liquid medium, they reached the same enzymatic activity as the strains grown on solid medium. Conversely, older *C. tropicalis* strains grown in liquid increased their phospholipase activity, remaining higher than in solid medium. For *C. parapsilosis*, the strains with poor phospholipase activity did not show any difference in activity at any time point, regardless the medium in which they were grown; on the contrary, the strains with high phospholipase activity demonstrated a higher enzymatic activity when they were grown on liquid medium than those grown on solid medium. For *C. glabrata* strains, no differences were observed in activity at any time point (Figure 4A and Supplementary Table S1). As a negative control, a *Trichosporon asahii* strain was used and as expected, we did not observe enzymatic activity at any time. An explanation for these results could be that the liquid medium increases the growth rate of the strains. To analyze this, we determined the fungi growth in each condition: interestingly, the growth of all *Candida* spp. strains was greater in solid medium than in liquid medium (Figure 4B and Supplementary Table S1).

**Figure 5:**
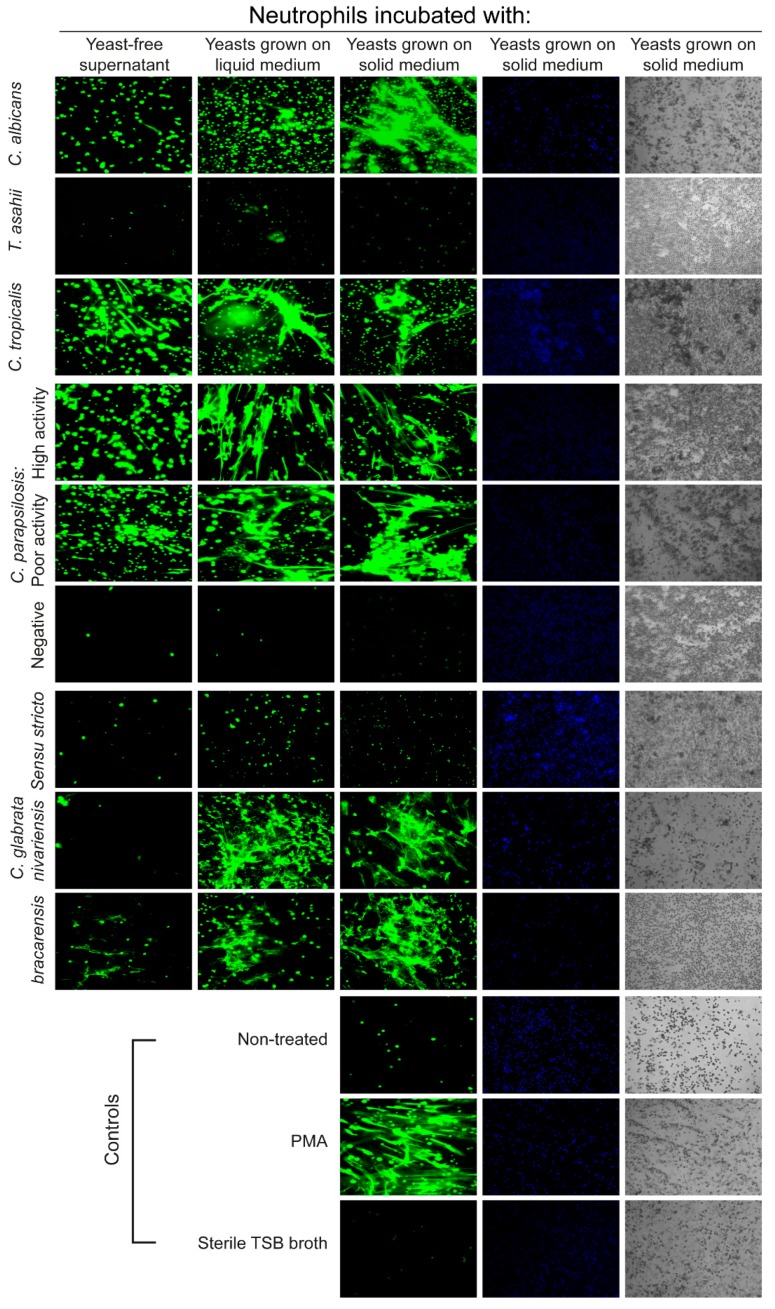
NETs formation correlates with expression of fungal phospholipases. Fluorescence microscopy images of non-treated neutrophils (NT), or treated with fungi and stained with Sytox to detect extracellular DNA after 180 min of treatment are shown. The treatments were: PMA (40 nM); yeast of *C. albicans*, *C. tropicalis*, *C. glabrata sensu stricto*, *C. bracarensis*, *C. nivariensis*; strains of *C. parapsilosis* with high, poor, or negative phospholipase activity; or phospholipase deficient *T. asahii* isolates grown on liquid or solid medium (MOI 0.2:1), fungi culture media filtered through 0.22 µM (1 μg/mL), sterile Trypticase Soy Broth (TSB) or left untreated. Additionally, bright field images and nuclei stained with Hoechst (blue) for the yeast grown in solid medium and controls are shown. All experiments were made in triplicate from two different donors each time.

We analyzed whether the phospholipases secreted by the *Candida* ssp. strains had the capacity to induce NETs formation. Yeast-free culture supernatants of *C. albicans*, *C. tropicalis*, and phospholipase positive strains of *C. parapsilosis*: all induced NETs formation. As expected, supernatants of the phospholipase-devoid isolates of *C. parapsilosis* and *T. asahii* did not induce NETs (Figure 5). To analyze whether additional fungal molecules could participate in NETs formation, we carried out analysis to determine hemolysin and DNAse production by our isolates, this may be important since it could be possible that our isolates could promote the death of neutrophils and the extrusion of DNA by the activity of hemolysin or that the absence of extracellular DNA observed for some strains could be the result of the activity of the fungal DNAses. We did not detect hemolysin activity in any of our isolates, however, a mild DNAse activity was found for all isolates with the exception of *Candida glabrata* (Supplementary Figure S2). A result that attracted our attention was that *C. glabrata* isolates, or their secreted products, did not induce NETs in any of the conditions tested (Figure 2B and Figure 5) although we found phospholipase activity on these strains (Figure 3, Figure 4, and Figure 5). We analyzed this aspect next.

### 3.4. NETs Formation in Candida glabrata

To determine if the inability to form NETs was a shared trait in other species of the *Candida glabrata* complex, we extended our analysis to include strains of *C. bracarensis* and *C. nivariensis*, two cryptic species belonging to this complex. First, we determined yeast morphology and phospholipase production in these species, and as expected, analyses showed the presence of only yeast forms with high phospholipase activity in the same way as *C. glabrata* sensu stricto (Supplementary Figure S3). On the other hand, NETs induction analyses demonstrated clear differences among the species within the complex, since *C. bracarensis* and *C. nivariensis* yeasts promoted DNA extrusion, whereas *C. glabrata* did not (Figure 5). The ability to induce NETs in our strains seems to be associated with factors present in the yeast, since their secreted products showed weak inducing activity.

## 4. Discussion

The induction of NETs has been observed in infections caused by different pathogens, however, an important role in response against opportunistic fungi has been recognized [2,32]. Size exclusion was proposed as a trait to promote NETs formation against fungi, since it was also shown that the hyphae of *C. albicans* induced NETs after being recognized by Dectin-1, while yeasts of the same species did not [11]. This suggests that NETs formation can be the result of an impossibility to eliminate fungi by phagocytosis but restrict infection. We analyzed if this constitutes a general response against other *Candida* ssp. and found robust formation induced by *C. albicans*, *C. parapsilosis*, and *C. tropicalis*, but not against *C. glabrata*. The lack of NETs in response against *C. glabrata* could be explained by the incapacity of these species to form large structures; although *C. tropicalis* and *C. parapsilosis* induced robust NETs formation even in the absence of filamentous structures. In line with our observations, other researchers have also found that NETs can be formed when yeasts are sensed to prevent fungal adhesion, invasion, and tissue destruction [12,15,33]. It is currently recognized that NETs formation represents an ordered process in which shortly after incubation of the neutrophils with NETs inductors, the nuclear membrane disrupts promoting the mix of the nuclear material with the granules in the cytoplasm, and later the plasmatic membrane breaks releasing the cell contents to the exterior rather than the unspecific and simultaneous rupture of the cell membranes that occurred in necrosis [7,34]. One observation that attracted our attention was the morphology of the different NETs induced by our isolates, since the morphology of the classic ‘fiber’ DNA was not always observed, and in its place only an expanded nuclear morphology was obtained for some strains. An explanation for this may lie in the stimulus used to initiate NETs formation, as was demonstrated by Kenny et al. In this paper, they show that—shortly after the incubation of neutrophils with various stimuli—different signaling pathways can be activated to induce NETs that can accelerate the process and can also influence its form (shown in the videos), since in some cases they do not lead to the fiber morphology observed after stimulation with PMA, and in its place a more "cloudy or globular" morphology is obtained [35]. We are currently working in analyzing the pathways initiated by our isolates to determine if different signaling pathways are activated. Another possibility would be that Sytox may stain the apoptotic cells in our analyses. To study this aspect, we carried out nuclear area quantification which allowed us to differentiate apoptotic cells from NETs since in the former the nucleus becomes piknotic (>80 µm^2^), and thus the nuclear area is smaller than in the normal neutrophils (80 µm^2^), while on NETs the nucleus delobulates but progressively increases its size (<80 µm^2^), in our experimental conditions all our isolates of *Candida* spp. induced an increment in the nuclear area correspondent to NETs formation.

Fungi components—such as β-glucan, secreted aspartic proteases, and mannans—are factors that are recognized by neutrophils through the Dectin-1 receptor to promote NETs formation [13,15]. However, other molecules such as fungal phospholipases could also be implicated in the process, since they are important for adherence, invasion, and cellular damage [16,21]. Analysis of phospholipases among our *Candida* ssp. isolates showed a heterogeneous production, activity, and secretion of PLA, PLB, PLC, and PLD not related to fungi growth, but influenced by metabolic changes due to environmental conditions. This is in line with diverse studies showing that, during growth and tissue invasion, pathogenic fungi can secrete DNAses, proteases, and phospholipases that allow them to reach diverse localizations within the host [18,36]. Additionally, the multi-purpose ability of these enzymes has been associated with its capacity to interact and promote modification in the cellular membranes; however, these could also lead to the initiation of a response when such changes are induced on immune cells. It is known that cPLA2 and PLB activity promotes formation of arachidonic acid from cellular membranes, which in turn activates cellular cyclooxygenase and lipoxygenase enzymes to secrete inflammation mediators such leukotrienes, thromboxanes, and prostaglandins, and cellular downstream pathways by PKC activation to control infections [17,37]. Likewise, phospholipase C can also induce cell membrane modifications promoting the formation of phosphatidylinositol biphosphate and diacylglycerol which in turn activates PKC in the cells [38,39]. PKC activation is central in NETs formation, as this molecule has been implicated directly in signaling pathways leading to neutrophil response [24,26,40].

Neutrophils incubated with the clinical isolates responded heterogeneously in NETs formation, since yeasts or secreted products of *C. albicans*, *C. tropicalis*, and some strains of *C. parapsilosis* were able to induce these structures; whereas no NETs were observed in yeasts or secreted products from *C. parapsilosis* or *T. asahii* strains that were negative in their phospholipase activity. From these observations, we propose that after infection, *Candida* ssp. strains produce phospholipases to promote their invasion, which in turn can be sensed by the neutrophils inducing NETs to possibly restrict the infection. Additional observations support this idea, as it has been described that these enzymes are overexpressed in planktonic forms of *C. albicans* and *Paracoccidioides brasiliensis*; however, when biofilms are formed, the phospholipases are downregulated [41,42,43]. This is important, since it has been shown that biofilms also inhibit NETs formation and fungal death mediated by these structures [14,33,44]. However, a possible confusion in the role of the phospholipases in NETs formation could also arise, as it has been previously reported that these enzymes can inhibit DNA extrusion in inflammatory conditions [45,46]. We think however, that it is important to consider the phospholipase origin, as the enzymes analyzed in these papers are of mammal origin and these are known to differ in function and structure from that of pathogen fungi in which their presence is important for increased virulence [16,47]. In our work, this is interesting, as we think that it could be related to the pathogenicity in our isolates, as all were obtained from septic patients and, therefore, their previous interaction with the immune system is highly probable. However, an important limitation in our study is that we did not use purified forms of the enzymes or fungi knock-out mutants. However, our results obtained with the phospholipase negative strains strongly suggest that these enzymes may have an important role.

Although we did not observe hemolysin production in our isolates and we propose that fungal phospholipases are important in NETs formation, we cannot rule out the possible implication of other fungal factors or their interaction, as other groups have described that secreted or purified aspartic proteases are able to induce NETs [15]. However, the role of these enzymes in NETs induction is still controversial, since it has been shown that these molecules are overexpressed when biofilms are formed [41,42,43], and it has been found that NETs are inhibited by biofilms [14,44], so the question of how biofilms inhibit the formation of NETs, ​​although aspartic proteases are overexpressed, still must be investigated. On the other hand, it is also known that pathogens are able to modulate their virulence factors according to the environment in which they are found [41]. Therefore, we believe that the induction of NETs could be modulated according to the virulence factors expressed by the pathogenic strains at the time of infection, implying greater complexity in the NETs formation response.

We also found that species in the *C. glabrata* complex possess different capacity to promote NETs, although all of our isolates showed phospholipase activity. We found that isolates of *C. glabrata* did not induce NETs, whereas *C. bracarensis* or *C. nivariensis* did. The importance of NETs in the response against fungi is underlined by the recent description of molecules present in these pathogens with the capacity to degrade and evade their antimicrobial activity and thus modulate the environment to avoid its elimination [48]. In line with this, it has been demonstrated that some isolates of *C. albicans* and *C. glabrata* sensu stricto can inhibit NETs formation through biofilm formation [33], which together with its high capacity of resistance against antifungal drugs, could be correlated with the high mortality rates of patients infected systemically with these strains [49]. An important perspective in our work is to demonstrate the mechanisms by which our isolates are preventing NETs formation, and the pathways involved in comparison with the cryptic species and with typed isolates.

In our paper, we contribute to the understanding of how neutrophils can initiate NETs formation after fungi recognition and provide strong correlative evidence that fungal phospholipases may contribute to promote DNA extrusion to form neutrophils. Thus, in addition to the recognition of the yeast forms and the sugars present in the cell wall, the phospholipases normally expressed in yeast and secreted to the environment by the fungi during the invasion processes may also contribute to promote NETs. We found that some clinical isolates may have differential capacity to promote NETs extrusion, which may explain the high mortality rates in patients infected with some of these species.

## Supplementary Materials

The following are available online at https://www.mdpi.com/2309-608X/5/2/28/s1, Figure S1. Precipitation halos of *Candida* ssp. clinical isolates grown in modified egg yolk agar. Percentages and forms of precipitation halos of *Candida* ssp., a total of 30 isolates of each species were analyzed and results are summarized. (A) *C. albicans*, (B) *C. tropicalis*, (C) *C. parapsilosis* (D), *C. glabrata*. (E) Representative images of halos produced by strains of *Candida* ssp., the forms were classified as follows: 1 marked, 2 Double halo, 3 Negative Strains without precipitation, 4 translucent halo with extended area (large halo), 5 translucent halo with minor area (small halo), 6 a strain of *Trichosporon asahii* was used as a negative control in which no halo of precipitation is observed; Figure S2. DNAse and Hemolisyn activity among isolates of *Candida* spp. (A) Experiments were made in triplicate and DNAse and hemolysin activity are summarized. (B) Representative images of DNAse and hemolysin activity in the *Candida* spp. isolates used in this study are shown. *Staphylococcus aureus* DNAse and hemolysin activity was used as positive control; Figure S3. Morphological characterization and phospholipase activity of *C. bracarensis* and *C. nivariensis*. (A) Bright field images depicting only yeast forms in culture (B) Phospholipase activity halos of each strains after growth in modified egg yolk agar; Table S1. Multiple comparison test analyses at different time points of phospholipase activity and growth for *Candida* ssp. species. Experiments were made by triplicate; ns not significant, **** *p* ≤ 0.0001, *** *p* ≤ 0.001, ** *p* ≤ 0.05, * *p* ≤ 0.5.
