# Supplementary material for: Candida albicans and non-albicans Isolates from Bloodstream Have Different Capacities to Induce Neutrophil Extracellular Traps
Source: J Fungi (Basel). 2019 Apr 1;5(2):28. doi: 10.3390/jof5020028 (PMC6616947; doi:10.3390/jof5020028)
Supplement: Supplementary file 1 [file jof-05-00028-s001.pdf]

Journal of Fungi

MDPI

Supplementary Information

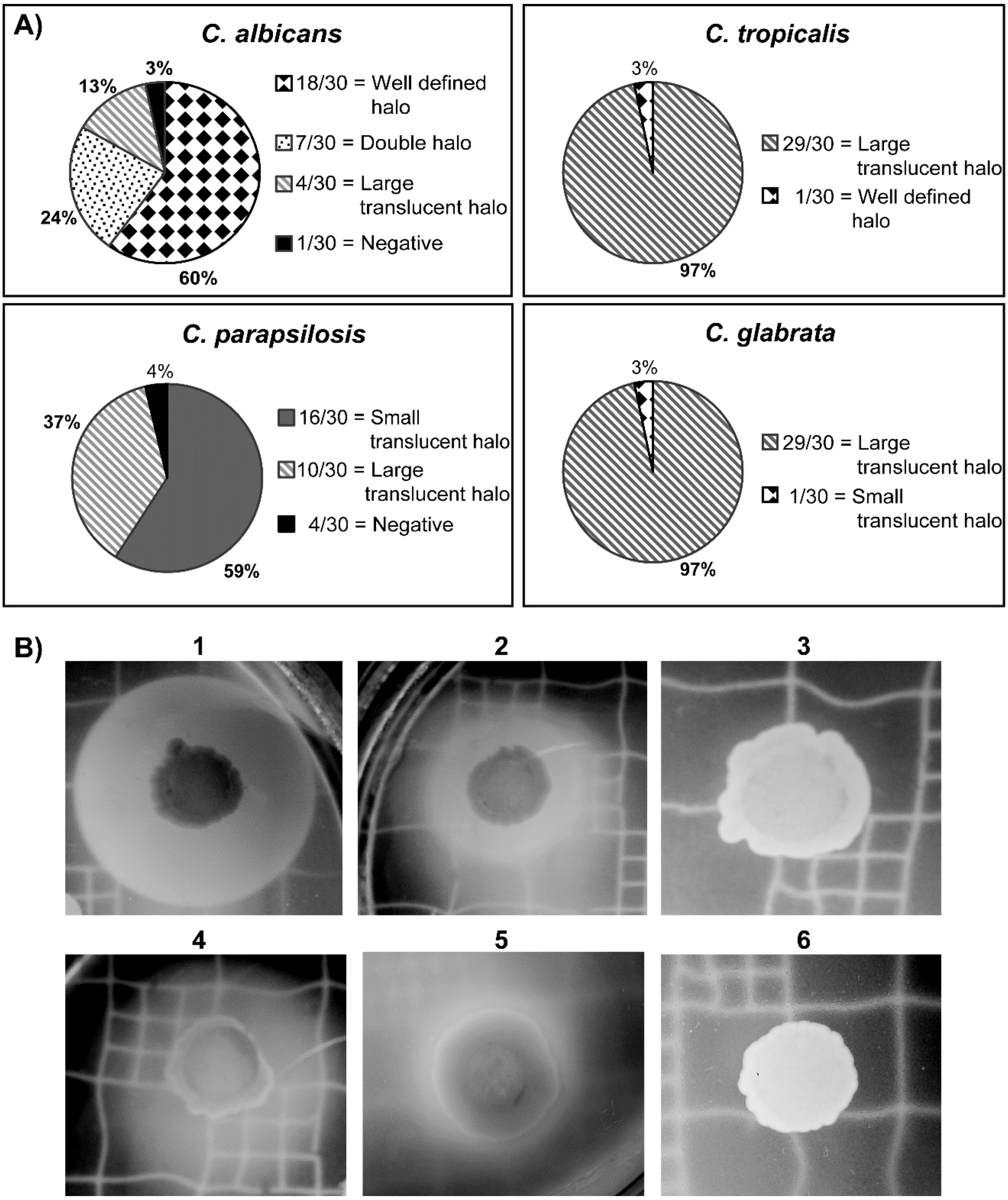

Figure 1. Precipitation halos of *Candida ssp.* clinical isolates grown in modified egg yolk agar. Percentages and forms of precipitation halos of *Candida ssp.*, a total of 30 isolates of each species were analyzed and results are summarized. (A) *C. albicans*, (B) *C. tropicalis*, (C) *C. parapsilosis* (D), *C. glabrata*. (E) Representative images of halos produced by strains of *Candida ssp.*, the forms were classified as follows: 1 marked, 2 Double halo, 3 Negative Strains without precipitation, 4 translucent halo with extended area (large halo), 5 translucent halo with minor area (small halo), 6 a strain of *Trichosporon asahii* was used as a negative control in which no halo of precipitation is observed.

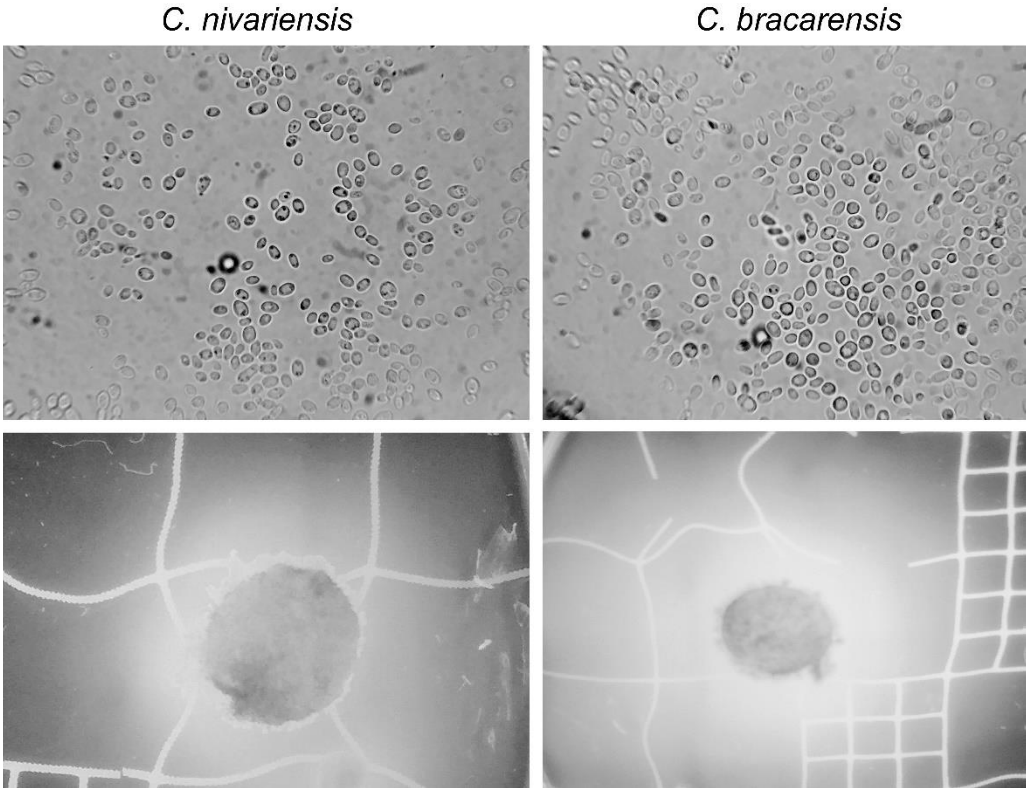

Figure 2. DNAse and Hemolisyn activity among isolates of *Candida spp.* (A) Experiments were made in triplicate and DNAse and hemolysin activity are summarized. (B) Representative images of DNAse and hemolysin activity in the *Candida spp.* isolates used in this study are shown. *Staphylococcus aureus* DNAse and hemolysin activity was used as positive control.

A)

Strain	Hemolysin activity	DNAses activity
C. albicans		
A1	-	+
A11	-	+
A13	-	+
A14	-	+
A25	-	+
C. tropicalis		
D2	-	+
D3	-	+
D14	-	+
D15	-	+
D24	-	+
C. parapsilosis		
B9 (negative phospholipase activity)	-	+
B4 (poor phospholipase activity)	-	+
B5 (poor phospholipase activity)	-	+
B11 (high phospholipase activity)	-	+
B22 (high phospholipase activity)	-	+
C. glabrata		
C12	-	-
C16	-	-
C20	-	-
C21	-	-
C26	-	-
C. bracarensis	-	-
C. nivariensis	-	-

No activity **-**
Weak activity **+**

B)

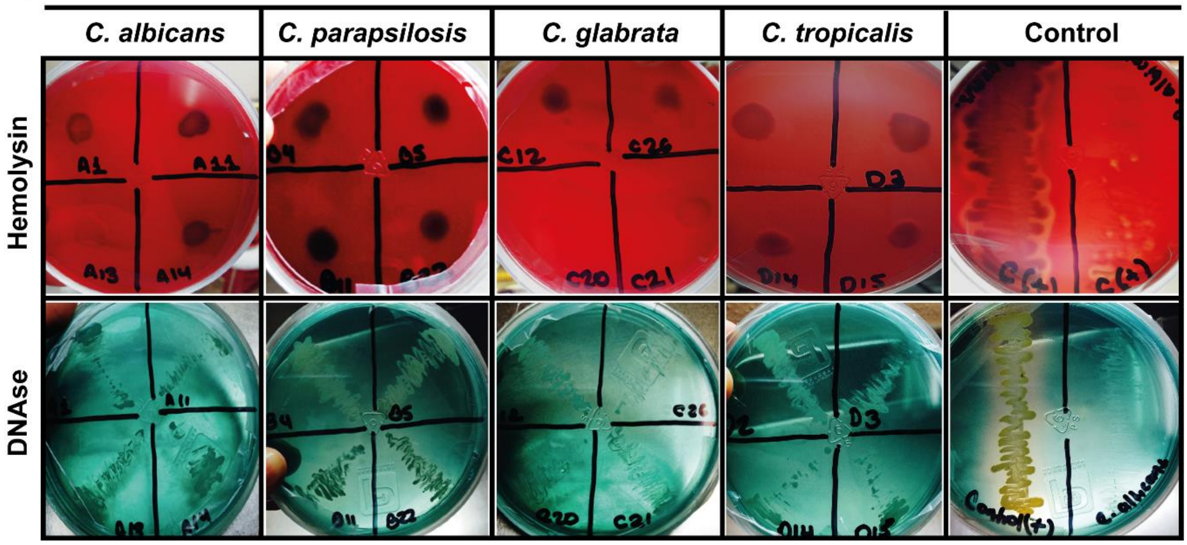

Figure 3. Morphological characterization and phospholipase activity of *C. bracarensis* and *C. nivariensis*. (A) Bright field images depicting only yeast forms in culture (B) Phospholipase activity halos of each strains after growth in modified egg yolk agar.

Table 1. Multiple comparison test analyses at different time points of phospholipase activity and growth for *Candida ssp.* species. Experiments were made by triplicate; ns not significant, **** $p \leq 0.0001$, *** $p \leq 0.001$, ** $p \leq 0.05$, * $p \leq 0.5$.

	24 h		48 h		72 h		120 h	
**Clinical Isolate and Treatment**	**Pz Growth value**		**Pz Growth value**		**Pz Growth value**		**Pz Growth value**	
**Solid media vs. Liquid pre-culture 24h**								
C. albicans	ns	****	ns	****	ns	****	ns	****
C. glabrata	*	ns	ns	****	ns	ns	ns	**
C. parapsilosis (++)	ns	*	ns	****	ns	****	ns	****
C. parapsilosis (++++)	**	ns	****	****	*	ns	**	***
C. tropicalis	ns	**	ns	ns	ns	***	ns	****
**Solid media vs. Liquid pre-culture 72h**								
C. albicans	ns	***	ns	***	ns	****	ns	****
C. glabrata	*	ns	ns	****	ns	ns	ns	**
C. parapsilosis (++)	ns	ns	ns	***	ns	****	ns	****
C. parapsilosis (++++)	*	**	****	****	ns	***	*	ns
C. tropicalis	ns	ns	ns	ns	ns	****	***	ns
**Solid media vs. Liquid pre-culture 120h**								
C. albicans	ns	ns	****	****	****	****	**	****
C. glabrata	ns	ns	ns	****	ns	***	ns	ns
C. parapsilosis (++)	ns	ns	ns	**	ns	****	ns	****
C. parapsilosis (++++)	ns	*	****	****	**	*	*	*
C. tropicalis	ns	ns	***	*	****	****	****	ns
**Solid media vs. *T. asahii***								
C. albicans	ns	****	****	****	****	ns	****	ns
C. glabrata	ns	*	****	****	****	ns	****	ns
C. parapsilosis (++)	ns	****	ns	ns	ns	ns	***	ns
C. parapsilosis (++++)	ns	****	ns	****	****	ns	****	ns
C. tropicalis	ns	****	****	****	****	ns	****	ns
**Liquid pre-culture 24h vs. Liquid pre-culture 72h**								
C. albicans	ns	ns	ns	ns	ns	ns	ns	ns
C. glabrata	ns	ns	ns	ns	ns	ns	ns	ns
C. parapsilosis (++)	ns	ns	ns	ns	ns	ns	ns	**
C. parapsilosis (++++)	ns	ns	ns	ns	ns	**	ns	*
C. tropicalis	ns	***	ns	ns	ns	**	**	****
**Liquid pre-culture 24h vs. Liquid pre-culture 120h**								
C. albicans	ns	*	****	ns	****	ns	*	ns
C. glabrata	*	ns	ns	ns	ns	ns	ns	ns
C. parapsilosis (++)	ns	ns	ns	ns	ns	ns	ns	***

C. parapsilosis (++++)	ns	ns	ns	ns	ns	ns	ns	****
C. tropicalis	*	ns	****	ns	****	****	****	****
**Liquid pre-culture 24h vs. *T. asahii***								
C. albicans	ns	ns	****	ns	****	****	****	****
C. glabrata	*	ns	****	ns	****	**	****	****
C. parapsilosis (++)	ns	ns	ns	*	*	****	***	****
C. parapsilosis (++++)	ns	****	****	ns	****	ns	****	****
C. tropicalis	****	****	***	***	***	***	****	***
**Liquid pre-culture 72h vs. Liquid pre-culture 120h**								
C. albicans	ns	ns	****	ns	****	ns	***	ns
C. glabrata	*	ns	ns	ns	ns	ns	ns	ns
C. parapsilosis (++)	ns	ns	ns	ns	ns	ns	ns	ns
C. parapsilosis (++++)	ns	ns	ns	ns	ns	ns	ns	***
C. tropicalis	ns	*	****	ns	****	ns	*	***
**Liquid pre-culture 72h vs. *T. asahii***								
C. albicans	ns	**	****	ns	****	****	****	****
C. glabrata	*	***	****	ns	****	**	****	****
C. parapsilosis (++)	ns	***	ns	ns	**	****	***	****
C. parapsilosis (++++)	**	**	****	ns	****	**	****	**
C. tropicalis	*	****	***	*	****	****	****	ns
**Liquid pre-culture 120h vs. *T. asahii***								
C. albicans	ns	****	ns	ns	****	****	****	****
C. glabrata	ns	***	****	ns	****	****	****	****
C. parapsilosis (++)	ns	****	ns	ns	ns	****	****	****
C. parapsilosis (++++)	*	****	****	ns	****	ns	****	ns
C. tropicalis	ns	****	****	ns	****	****	****	****
